# Evaluation of the Effects of Some Brazilian Medicinal Plants on the Production of TNF-***α*** and CCL2 by THP-1 Cells

**DOI:** 10.1155/2015/497123

**Published:** 2015-03-23

**Authors:** Grasielle S. Gusman, Priscilla R. V. Campana, Luciano C. Castro, Rachel O. Castilho, Mauro M. Teixeira, Fernão C. Braga

**Affiliations:** ^1^Department of Pharmaceutical Sciences, Faculty of Pharmacy, Universidade Federal de Minas Gerais, Avenida Antônio Carlos 6627, Campus Pampulha, 31.270-901 Belo Horizonte, MG, Brazil; ^2^Department of Biochemistry, Institute of Biological Sciences, Universidade Federal de Minas Gerais, Avenida Antônio Carlos 6627, Campus Pampulha, 31.270-901 Belo Horizonte, MG, Brazil

## Abstract

Several plant species are traditionally used in Brazil to treat various inflammatory diseases. Tumor necrosis factor- (TNF-) *α* and chemokine (C-C motif) ligand 2 (CCL2) are key inflammatory mediators in diseases like rheumatoid arthritis and atherosclerosis, respectively; nevertheless, only a few extracts have been assayed against these targets. We herein report the effect of 19 plant extracts on TNF-*α* and CCL2 release by lipopolysaccharide- (LPS-) stimulated THP-1 cells, a human monocytic leukemia cell line, along with their radical scavenging activity on DPPH. The extracts of *Caryocar brasiliense*, *Casearia sylvestris*, *Coccoloba cereifera*, and *Terminalia glabrescens* inhibited TNF-*α* production in a concentration-dependent manner. Fractionation of these extracts potentiated the anti-TNF-*α* effect, which was shown to concentrate in polar fractions, mainly composed by polyphenols. Significant CCL2 inhibition was elicited by *Lippia sidoides* and *Terminalia glabrescens* extracts, whose fractionation resulted in highly active low polar fractions. All assayed extracts showed strong radical scavenging activity, but antioxidant activity did not correlate with inhibition of TNF-*α* or CCL2 production. Our results allowed identifying extracts with selective capacity to block cytokine production; therefore, further purification of these extracts may yield molecules that could be useful in the treatment of chronic inflammatory diseases.

## 1. Introduction

Inflammation is a complex physiological response of vascularized tissues to noxious stimuli as pathogens, damaged cells, or irritating agents. Although it is primarily a physiological and beneficial process, nonresolved inflammation can be involved in the pathogenesis and progression of several inflammatory diseases, including rheumatoid arthritis (RA) and cardiovascular diseases (CD) [1, 2].

The tumor necrosis factor- (TNF-) *α* is a key proinflammatory cytokine that mediates apoptosis, inflammation, and immunity and is believed to play a role in various chronic inflammatory diseases including RA, psoriasis, inflammatory bowel disease (IBD), diabetes, cancer, and sepsis [3–5]. Indeed, anti-TNF-*α* therapy is currently in clinical use for management of RA and IBD. Nevertheless, treatment with biological agents is costly for low income countries and their use may be associated with adverse effects and predisposition to malignancies, especially lymphoma [6, 7].

The chemokine (C-C motif) ligand 2 (CCL2) or chemokine monocyte chemoattractant protein- (MCP-) 1 is believed to play a major role in the pathogenesis of hypertensive vascular disease and atherosclerosis [8, 9] and may also contribute to the pathogenesis of RA, idiopathic pulmonary fibrosis, and tumors [10]. There appears to be a link between inflammatory processes and hypertension, wherein vascular inflammation plays an important role in the pathogenesis and progression of hypertension [11, 12]. Inflammation participates in all stages of the atherosclerotic process, including endothelial dysfunction, monocyte adhesion and penetration, plaque formation and progression, and plaque rupture and thrombosis [11]. CCL2 contributes to the atherosclerotic process as it strengthens the adhesion and promotes monocytes to transmigrate into intima and later mature into macrophages [13].

The imbalance of reactive oxygen species (ROS) generation is recognized to aggravate inflammation. In the last years, considerable attention has been given to the participation of ROS in the pathogenesis of RA and hypertension [14]. ROS such as superoxide anion (O_2_^•−^), hydrogen peroxide (H_2_O_2_), and hydroxyl radicals (^•^OH) can initiate and perpetuate the inflammatory cascade causing additional tissue damage via further activation of NF-*κ*B [15]. On the other hand, there is also evidence to suggest that ROS can reduce the inflammatory process or induce resolution of inflammation [16]. Therefore, ROS seem to play a complex role in inflammation and temporal control of ROS generation can determine their action as pro- or anti-inflammatory agents [2].

Brazil is considered to have the richest biota among the megadiversity countries, possessing 20–22% of all existing plants and microorganisms [17]. Several plant species are traditionally used in the country to treat different inflammatory conditions. A review of Falcão and collaborators listed 75 plants that have been investigated in the country for their* in vivo* anti-inflammatory properties [18]. In spite of its rich plant biodiversity and cultural diversity of traditional populations, only a limited number of plant products from Brazil have reached the markets in industrialized countries [19]. Therefore, the investigation of biological properties of Brazilian medicinal plants offers a unique opportunity for drug development.

Continuous studies on the molecular basis of inflammatory processes resulted in the identification of key targets to be exploited for drug development and allowed the investigation of plant extracts in different cell-based assays [20, 21]. Besides the identification of anti-inflammatory plants, this approach also discloses possible cytotoxic effects and furnishes clues on their mechanism of action. Within this context, we have focused our investigation on the effects of Brazilian plants on the production of TNF-*α* and CCL2 release by THP-1 cells stimulated by LPS. TNF-*α* and CCL2 are key mediators in chronic inflammatory processes like arthritis and atherosclerosis and the plants were selected based on their traditional use to treat these diseases or other inflammatory conditions. We have also investigated the antioxidant properties of the plants using the DPPH assay, since oxidative stress may also contribute to the pathogenesis of arthritis and atherosclerosis.

## 2. Material and Methods

### 2.1. Plant Materials, Extracts Preparation, and Fractionation

A total of 18 plant species were selected for study based on their traditional uses in Brazil to treat different inflammatory processes (Table 1).

The species were identified by botanists from the Botanical Department, Instituto de Ciências Biológicas, UFMG, Belo Horizonte, Brazil, where voucher specimens are deposited (see Table 1 for voucher numbers). After drying at 45°C, during 72 h, the plant material was powdered and extracted by exhaustive percolation with ethanol 96 GL at room temperature (Table 2). The solvent was removed in a rotatory evaporator at 50°C under reduced pressure, to furnish the crude extracts (see Table 2 for extraction yields). Portions (2 g) of the extracts of the most active plants were dissolved in methanol/water (3 : 7) and sequentially partitioned with equal volumes (3 × 50 mL) of* n*-hexane, dichloromethane, and ethyl acetate. Solvents were removed in a rotatory evaporator, at maximum temperature of 50°C, to afford the* n*-hexane, DCM, EtOAc, and water fractions.

### 2.2. Cell Line and Assay of Inflammatory Mediators

The anti-inflammatory activity of the extracts and fractions was evaluated by measuring TNF-*α* and CCL2 produced by LPS-stimulated THP-1 cells employing an immunoassay [22]. THP-1 cells (ATCC TIB-202) were cultivated in RPMI 1640 medium (Sigma, USA) supplemented with 0.05 mM 2-mercaptoethanol, 10% FBS (Gibco, USA), 100 U/mL of penicillin (Sigma Aldrich, USA), and 100 *μ*g/mL of gentamicin (Sigma Aldrich, USA) at 37°C in an atmosphere containing 5% CO_2_.

The medium was renovated twice a week, when cell concentrations reached 1.0 × 10^6^ cells/mL. The cells were transferred to a 96-well microplate at a density of 100,000 cells per well, incubated for 18 h, and pretreated with the samples for 3 h. LPS (Sigma Aldrich, USA) was administrated at 100 ng/mL as inflammatory stimulus. After incubating the plate at 37°C overnight, it was centrifuged (1800 g, 5 min, 16°C), the supernatant was collected, and TNF-*α* was measured using the cytokine-specific sandwich quantitative enzyme-linked immune-sorbent assay (ELISA) according to the manufacturer's instructions (TNF-*α* Duo Set and CCL2 Duo Set, R&D Systems, USA). Cell viability was evaluated for all tested extracts, at the highest assayed concentration (250 *μ*g/mL), by the MTT method [23], using untreated cells as reference for viability. Samples that gave cell viability higher than 90% were considered nontoxic for the THP-1 cell line. The percentages of TNF-*α* and CCL2 inhibition were calculated by the ratio between the amount of mediators secreted by treated cells (pg/mL) and their baseline level (pg/mL) observed for solvent control (0.1% DMSO). The inhibitory effects on TNF-*α* and CCL2 release by LPS-activated THP-1 cells of the crude extracts and fractions were evaluated at 62.5, 125, and 250 *μ*g/mL. Dexamethasone (Sigma Aldrich, USA) was employed as positive control (0.1 *μ*M). For the assays, all samples were solubilized in RPMI medium containing 0.1% DMSO.

### 2.3. DPPH (2,2-Diphenyl-1-picrylhydrazyl) Assay

The DPPH radical scavenging assay was performed according to a previously reported method [24]. Briefly, samples solutions prepared in methanol (0.5–7.5 *μ*g/mL) or pure methanol (control) were transferred to a 96-well microplate containing 70 *μ*mol/L DPPH (Sigma Aldrich, USA) methanolic solution. The plate was shaken vigorously for 1 min and incubated in the dark for 30 min, at 37°C, following absorbance reading at the wavelength of 517 nm, using a multiwell scanning spectrophotometer (Tecan Infinity M200, Switzerland). The radical scavenging capacity (expressed as percentage) was calculated as the rate between (Abs_control_ − Abs_sample_)/Abs_control_ × 100. Rutin (Sigma Aldrich, USA) was employed as positive control.

### 2.4. Statistical Analysis

The IC_50_ values were determined for the extracts which inhibited more significantly the production of TNF-*α* and CCL2 by THP-1 cells, as well as for all extracts evaluated in the DPPH radical scavenging assay. In each case, IC_50_ values were calculated using at least five concentrations and represent the mean ± SD for *n* = 5. The inhibition values of inflammatory mediators were compared to the control cells stimulated with LPS using analysis of variance (one-way ANOVA) followed by Newman-Keuls post test. For the DPPH assay, the data were compared by one-way ANOVA, followed by Tukey's test [25], representing the mean ± SD for *n* = 5. *P* values less than 0.05 (*P* < 0.05) were considered significant.

## 3. Results

### 3.1. Effects on LPS-Induced Proinflammatory Mediators TNF-*α* and CCL2

The ethanol extracts of the selected plants were initially evaluated on THP-1 cells at 250 *μ*g/mL to investigate cellular toxicity. All extracts produced cell viability above 90%, being considered noncytotoxic and evaluated in the subsequent experiments. The inhibition of TNF-*α* and CCL2 release by LPS-stimulated THP-1 cells was assayed at three concentrations to investigate concentration-dependent response in the biological effect.

Among the 19 extracts assayed, those of* Caryocar brasiliense *(barks and leaves),* Casearia sylvestris*,* Chrysophyllum marginatum*,* Coccoloba cereifera*, and* Terminalia glabrescens* significantly inhibited TNF-*α* production by THP-1 cells in a concentration-dependent manner (Table 2). Other extracts were active at specific concentrations, but the response could not be characterized as concentration-dependent, including* Barbacenia flava*,* Lippia sidoides*,* Sida carpinifolia*,* Sida glaziovii*, and* Terminalia catappa*. On their turn, the extracts of* Calophyllum brasiliense* and* Erythroxylum tortuosum* increased TNF-*α* level at all assayed concentrations, suggesting that they may have proinflammatory effects. The remaining extracts showed variable responses, either enhancing or decreasing TNF-*α* levels, depending on the assayed concentration, suggesting nonspecific responses (Table 2).

The most active extracts had their IC_50_ values determined and were found to be in the range of 124.0 ± 1.0 to 224.0 ± 1.0 *μ*g/mL, respectively, for* Casearia sylvestris* and* Caryocar brasiliense *leaves (Table 3). The extracts of* Caryocar brasiliense* (barks) and* Chrysophyllum marginatum* did not reach the plateau for IC_50_ determination, indicating that higher concentrations were needed.

Among the 19 extracts evaluated, only those of* Lippia sidoides* and* Terminalia glabrescens* inhibited significantly CCL2 release by LPS-stimulated THP-1 cells in a concentration-dependent manner (Table 2).* Lippia sidoides* reduced CCL2 production by 96.0 ± 0.1% when tested at 250 *μ*g/mL, indicating potency higher than the positive control dexamethasone.* Casearia sylvestris* was also very potent and had a very steep inhibition curve as inhibition of chemokine production was already maximal at 125 *μ*g/mL. Most of the assayed extracts elicited CCL2 inhibition at one single concentration, mostly at 250 *μ*g/mL, whereas increase in chemokine concentration was observed at the medium and intermediate concentrations. The IC_50_ values were determined only for the extracts of* Lippia sidoides* (64.2 ± 1.0 *μ*g/mL) and* Terminalia glabrescens* (189.4 ± 1.0 *μ*g/mL), since the remaining 17 extracts did not inhibit CCL2 release in a concentration-dependent manner and the responses were very steep for* Casearia sylvestris*.

**Figure 1:**
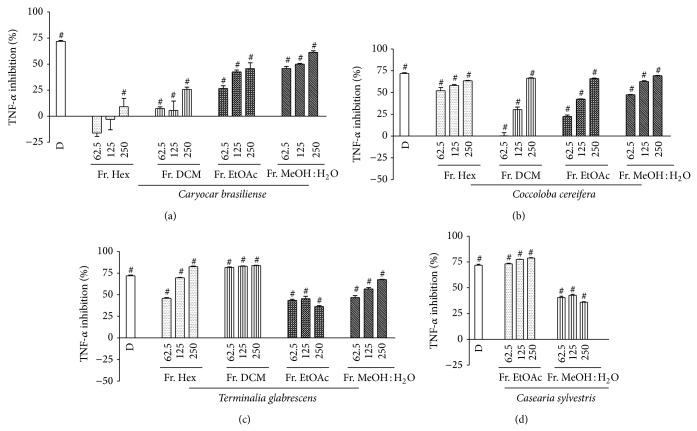
Effects of the fractions from Brazilian plants on TNF-*α* release by LPS-activated THP-1 cells, assayed at different concentrations (62.5, 125, and 250 *μ*g/mL): (a)* Caryocar brasiliense*, (b)* Coccoloba cereifera*, (c)* Terminalia glabrescens*, and (d)* Casearia sylvestris*. Data represent the mean inhibition (% ± SD) from three separate experiments. Differences in TNF-*α* release elicited by the fractions were analyzed by one-way ANOVA followed by multiple comparisons of Newman-Keuls. In each fraction, # indicates significant inhibition of TNF-*α* release in comparison to LPS-stimulated cells (*P* < 0.05). D = dexamethasone (0.1 *μ*M; positive control). TNF-*α* mean value of LPS-stimulated THP-1 cells was 1092.49 ± 4.87 pg/mL and the basal level was 52.54 ± 1.21 pg/mL.

**Figure 2:**
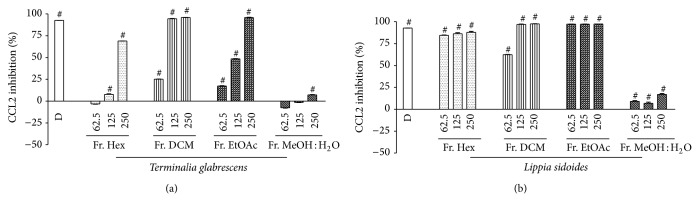
Effects of the fractions from Brazilian plants on CCL2 release in LPS-activated THP-1 cells, assayed at different concentrations (62.5, 125, and 250 *μ*g/mL): (a)* Terminalia glabrescens* and (b)* Lippia sidoides*. Data represent the mean inhibition (% ± SD) from three separate experiments. Differences in CCL2 release elicited by the fractions were analyzed by one-way ANOVA followed by multiple comparisons of Newman-Keuls. In each fraction, # indicates significant inhibition of CCL2 release in comparison to LPS-stimulated cells (*P* < 0.05). D = dexamethasone (0.1 *μ*M; positive control). CCL2 mean value of LPS-stimulated cells was 1388.15 ± 3.43 pg/mL and the basal level was 58.97 ± 0.69 pg/mL.

The extracts which elicited potent inhibition of the proinflammatory mediators, respectively,* Caryocar brasiliense* (leaves),* Casearia sylvestris*, and* Coccoloba cereifera* for TNF-*α*,* Lippia sidoides* for CCL2, and* Terminalia glabrescens* for both, were fractionated by partition between immiscible solvents and the resulting fractions had their anti-inflammatory effect assayed. In general, the anti-TNF-*α* activity was mainly found in the ethyl acetate and water fractions (Figure 1), whereas low polarity fractions of* Terminalia glabrescens* were also active. Interestingly, all fractions of this species promoted significant CCL2 inhibition, mostly the low and medium polar fractions (Figure 2). A similar result was found for* Lippia sidoides*, whose CCL2 activity concentrated in the hexane, DCM, and EtOAc fractions (Figure 2). These findings suggest that CCL2 inhibition is probably elicited by low and medium polar compounds, whereas high polar constituents may account for the anti-TNF-*α* effect of the assayed extracts.

### 3.2. Antioxidant Activity

The antioxidant activity of the extracts was investigated* in vitro* using the DPPH assay and the IC_50_ values are listed in Table 3. The ratio between the IC_50_ values of the extracts and rutin (positive control) was calculated and allowed categorizing the extracts as high (ratio ≤ 0.5), intermediate (ratio between 0.51 and 0.99), and low (ratio ≥ 1) active. Based on this ranking, the highest DPPH free radical scavenging activities were elicited by the extracts of* Caryocar brasiliense* (leaves),* Coccoloba cereifera*, and* Paepalanthus bromelioides*. On their turn, the extracts of* Terminalia glabrescens*,* Erythroxylum gonocladum*, and* Sida carpinifolia* showed antioxidant properties less potent than rutin, whereas the other extracts were active, with IC_50_ values lower than rutin. Interestingly, among the three extracts with the highest radical scavenging activity, those of* Caryocar brasiliensis* and* Coccoloba cereifera* also elicited significant inhibition of TNF-*α*, suggesting a correlation between the activities, which was also found for other active extracts. On the other hand, none of the extracts with low IC_50_ values in the DPPH assay inhibited CCL2 significantly. It should be also mentioned that the extract of* Terminalia glabrescens* significantly inhibited both proinflammatory mediators, but its antioxidant activity was lower than rutin. This finding suggests that compounds other than polyphenols may account for the anti-inflammatory properties of this extract.

## 4. Discussion

TNF-*α* is a potent proinflammatory cytokine detected in high concentrations in the synovial cavity of patients with rheumatoid arthritis [26]. It is secreted by activated monocytes and subsequently activates other cell types in the blood vessel and thus initiates/promotes the progression of atherosclerosis [27]. CCL2 is a chemokine responsible for monocytes and macrophages migration to inflammatory sites, which also has a direct effect on angiogenesis [28–30]. Increased levels of CCL2 were found to correlate with complications associated with atherosclerosis, including ischemic stroke, myocardial infarction, and cardiovascular disease mortality [31–33].

There is much evidence to suggest the participation of inflammatory processes in the basis of hypertension, suggesting a link between inflammation and hypertension. Moreover, vascular inflammation plays an important role in hypertension genesis and progression, resulting in aggravation of atherosclerosis [11, 12]. Previous studies have demonstrated high levels of CCL2 in patients with arterial hypertension, as well as increased levels of TNF-*α* on DOCA-salt models [34, 35]. Therefore, the identification of TNF-*α* and CCL2 antagonists from natural sources would represent a new approach for the management of various chronic inflammatory conditions, including rheumatoid arthritis and cardiovascular diseases like arthrosclerosis [26, 36].

The screening of plant extracts with anti-inflammatory properties on a cell-based assay platform, targeting key inflammatory mediators as TNF-*α* and CCL2, is recognized nowadays as a valid strategy to select plant species for phytochemical studies [20, 21]. Most of the reported screenings have been carried out at one single concentration. This approach impairs to investigate concentration-dependent response in the biological effect and consequently to eliminate the extracts with unspecific effect for further studies. Therefore, in this study we assayed the extracts at three levels and the most active ones had their IC_50_ values determined.

The plants described here were selected based on their traditional use to treat inflammatory and/or cardiovascular diseases by the species themselves or others of the same genus. Respectively, six and two extracts among a total of 19 inhibited significantly TNF-*α* and CCL2 release by LPS-stimulated THP-1 cells, in a concentration-dependent manner, indicating the validity of the approach employed for plant selection. There are not many reports on the screening of plant extract targeting these inflammatory mediators. Among four plant extracts screened by Nishijima [37], only* Rhamnidium elaeocarpum* elicited significant anti-TNF-*α* activity in LPS-stimulated murine peritoneal macrophages, but at higher concentration (2 mg/mL; 48.86% inhibition) than that employed in our study. On its turn,* Hypericum triquetrifolium* inhibited TNF-*α* release by LPS-stimulated THP-1 cells in a concentration-dependent manner, in a similar range of concentrations reported here [38].

Other studies disclosed plant extracts with similar or even higher TNF-*α* inhibitory activity. Extracts of different polarities from nine medicinal plants from Thailand showed anti-TNF-*α* effects in LPS-stimulated human monocytes, with IC_50_ values ranging from 0.28 to >50 *μ*g/mL [21]. In another study with 64 medicinal plants from South-East Spain, extracts of* Bupleurum fruticosum*,* Chamaespartium tridentatum*,* Genista ramosissima*,* Helichrysum stoechas*,* Mercurialis tomentosa*,* Ononis ramosissima*,* Peganum harmala*,* Picnomon acarna*,* Retama sphaerocarpa*, and* Santolina viscosa* were active at inhibiting TNF-*α* at 10 *μ*g/mL in LPS-stimulated human monocytes [20].

Regarding the anti-inflammatory effect on CCL2, our study revealed two species,* Casearia sylvestris* and* Lippia sidoides*, with inhibition rates above 95% at the highest tested concentration. The effect of other plant extracts or isolated compounds on this chemokine has been previously investigated in THP-1 cells. The administration of a decoction from* Rehmannia glutinosa* rhizomes to THP-1 cells treated with AGEs downregulated the expression of proinflammatory genes, including CCL2 mRNA levels, when tested at 500 and 1000 *μ*g/mL [39]. Similar results were reported for alcoholic and aqueous extracts from* Ocimum sanctum* leaves, which markedly inhibited the expression of proinflammatory genes, CCL2 marker including, when assayed at 1000 *μ*g/mL in LPS-stimulated THP-1 cells [27]. On its turn, catalpol, an iridoid glucoside isolated from the roots of* Rehmannia glutinosa*, reduced the expression of proinflammatory mediators such as CCL2, TNF-*α*, iNOS, and RAGE, when administrated at 300 and 500 *μ*g/mL to AGE-stimulated THP-1 cells [40]. These data demonstrate unequivocally the anti-inflammatory potential of the plant species disclosed in the present study, which inhibited CCL2 at much lower concentrations.

The HPLC-DAD fingerprints recorded for the most active extracts (*Caryocar brasiliense *leaves*, Casearia sylvestris*,* Coccoloba cereifera*,* Terminalia glabrescens*, and* Lippia sidoides*) indicate they are majorly composed by polar compounds, mainly polyphenols like flavonoids and proanthocyanidins (data not shown). Excepting* Coccoloba cereifera* and* Lippia sidoides*, the occurrence of flavonoids and proanthocyanidins has been described for all the above-mentioned species [41–44]. On the other hand, flavonoids have been isolated from other* Coccoloba* and* Lippia* species [45, 46].

Some of the active species reported here have had their anti-inflammatory effect previously investigated, mostly for their essential oils, such as* Lippia sidoides* [47, 48],* Caryocar brasiliense* [49], and* Casearia sylvestris* [50]. The anti-inflammatory effect of* Terminalia glabrescens* and* Coccoloba cereifera* is reported here for the first time.

The obtained results suggest that polar compounds may be responsible for the TNF-*α* inhibition elicited by the species, since the activity was concentrated in the ethyl acetate and methanolic aqueous fractions. In general, these fractions showed higher inhibition rates than the crude extracts, indicating that the anti-inflammatory effect is potentiated by fractionation. On the other hand, higher CCL2 inhibitory activities were observed for the low polar fractions. This finding indicates that different classes of compounds may account for the inhibition of TNF-*α* and CCL2, suggesting a selective effect.

Significant DPPH scavenging activity was observed for all tested extracts, probably due to the presence of phenolic compounds, whose antioxidant properties have been extensively reported using* in vivo* and* in vitro* assays [51, 52]. ROS seems to play a complex role in inflammation. According to Alessandri and coworkers [2], a small concentration of ROS can promote an improvement in the resolution of inflammatory processes. On the other hand, the uncontrolled increase of ROS generation can initiate and perpetuate inflammatory cascades, causing an additional damage through the regulation of genes involved in inflammatory responses [15]. Therefore, a temporal control of ROS generation can determine their action as pro- or anti-inflammatory agents [2]. Considering that rheumatoid arthritis is a chronic disease, antioxidant properties allied to TNF-*α* and CCL2 inhibition might be beneficial for inflammation resolution.

Taken together, our results allowed identifying four extracts with selective anti-inflammatory response, three with marked anti-TNF-*α* activity and one with CCL2 inhibiting effect, in addition to one extract active against both mediators. Interestingly, the active extracts showed more selectivity for TNF-*α*, whereas those inhibiting CCL2 in a greater extension also presented anti-TNF-*α* effect. A selective anti-TNF-*α* effect is desirable for developing new antirheumatic drugs, since the administration of CCL2 monoclonal antibody to patients with rheumatoid arthritis did not ameliorate the disease and even aggravated the symptoms at higher doses. The effect was related to a dramatic increase of antibody-complexed CCL2 levels in peripheral blood [33, 53]. On the other hand, selective CCL2 inhibition indicates a new approach for treating atherosclerosis.

## 5. Conclusion

Plant screening directed towards a disease specific mediator is undoubtedly a further step in the quest for new active natural products. Some of the extracts evaluated in this study were able to reduce TNF-*α* release selectively, whereas others inhibited preferably CCL2, pointing out the first group of plants as potential sources of compounds for management of rheumatoid arthritis, whereas the second ones would be more suited for the treatment of cardiovascular diseases like atherosclerosis. Moreover, all assayed extracts showed radical scavenging properties, which may have additional beneficial effects for treating inflammation related diseases.

## Figures and Tables

**Table 1:** Ethnopharmacological uses and collection and identification data of the plant species selected for study.

Plant species	Family	Location	Voucher number	Ethnopharmacological uses	References
*Barbacenia flava* Martius ex Schultes f.	Velloziaceae	Serra do Cipó	BHCB 144535	Skin lesions	[54]
*Calophyllum brasiliense *Camb.	Clusiaceae	Perdizes	BHCB 27720	Inflammation, diabetes, hypertension	[55, 56]
*Caryocar brasiliense *Camb.	Caryocaraceae	Montes Claros	HMC 338	Asthma, bronchopulmonary diseases	[57–59]
*Casearia sylvestris *Sw.	Salicaceae	Montes Claro	HMC 3008	Inflammation, cancer, antibacterial	[60–63]
*Chrysophyllum marginatum *(Hook. & Arn.)	Sapotaceae	UFMG campus, Belo Horizonte	BHCB 23319	Cognitive disorders, coronary diseases, atherosclerosis, and cancer	[64, 65]
*Coccoloba cereifera *Schwan.	Polygonaceae	Serra do Cipó	BHCB 73215	*Coccoloba * species: skin rash, ulcer, and renal diseases	[66, 67]
*Cuphea carthagenensis* (Jacq.) J.f. Macbr	Lythraceae	Perdizes	BHCB 25816	Cardiovascular diseases, atherosclerosis	[68, 69]
*Erythroxylum gonocladum *(Mart.) O. E. Schulz	Erythroxylaceae	Serra da Piedade	BHCB 118812	Inflammation, bronchitis, and asthma	[70, 71]
*Erythroxylum suberosum *St. Hil.	Erythroxylaceae	Lagoa Santa	BHCB 111.068	Inflammation, bronchitis, and asthma	[70, 71]
*Erythroxylum tortuosum* Mart.	Erythroxylaceae	Lagoa Santa	BHCB 111.065	Inflammation, bronchitis, and asthma	[70, 71]
*Lippia sidoides *Cham.	Verbenaceae	Montes Claros	HMC 843	Fungal infections, inflammation, burns	[46, 72]
*Paepalanthus bromelioides *Silv.	Eriocaulaceae	Serra do Cipó	BHCB 24241	Cytotoxic, antimutagenic	[73, 74]
*Phoenix roebelenii *O'Brien	Arecaceae	UFMG campus, Belo Horizonte	BHCB 130070	Diuretic	[75]
*Sida carpinifolia *(L.f.) K. Schum.	Malvaceae	Itabira	BHCB 65229	Bronchitis, depression, fever	[76]
*Sida glaziovii *K. Schum.	Malvaceae	UFMG campus, Belo Horizonte	BHCB 34621	* Sida *species: diarrhea, intestinal problems, infections, inflammation	[77]
*Terminalia catappa *L.	Combretaceae	UFMG campus, Belo Horizonte	BHCB 47160	Cardiovascular diseases, hypertension	[78, 79]
*Terminalia glabrescens *Mart.	Combretaceae	UFMG campus, Belo Horizonte	BHCB 130413	Cancer, infections	[80, 81]
*Vellozia piresiana *L.B.Sm.	Velloziaceae	Serra do Cipó	BHCB 43713	*Vellozia *species:anti-inflammatory and antirheumatic	[82]

**Table 2:** Extraction yields and effect of the ethanol extracts from Brazilian plants on TNF-*α* and CCL2 release by LPS-stimulated THP-1 cells.

Plant species	Plant part	Extraction yield (%)	Concentration (*μ*g/mL)	TNF-*α* inhibition (% ± SD, *n* = 3)	CCL2 inhibition (% ± SD, *n* = 3)
Control			LPS (100 ng)	1030.4 ± 2.7^a^	1193.4 ± 9.1^a^
			DMSO (0.1%)	50.3 ± 1.1^a^	48.1 ± 1.0^a^
			Dexamethasone (0.1 *μ*M)	90.7 ± 2.2^*^	87.7 ± 0.6^*^

*Barbacenia flava *	Aerial parts	8.3	62.5	27.5 ± 7.5^*^	−16.3 ± 6.9
			125	36.6 ± 0.6^*^	−10.6 ± 12.7
			250	45.4 ± 12.9^*^	38.4 ± 8.0^*^

*Calophyllum brasiliense *	Aerial parts	8.0	62.5	−48.2 ± 8.7	−15.1 ± 5.8
			125	−29.0 ± 4.4	−9.6 ± 5.2
			250	−93.8 ± 6.4	23.7 ± 3.4^*^

*Caryocar brasiliense *	Leaves	39.4	62.5	19.4 ± 0.5^*^	−15.5 ± 5.8
			125	41.3 ± 0.8^*^	−8.1 ± 5.4
			250	61.1 ± 3.9^*^	4.6 ± 4.7

*Caryocar brasiliense *	Barks	29.0	62.5	17.6 ± 4.2^*^	9.1 ± 9.8
			125	43.7 ± 2.0^*^	0.2 ± 4.3
			250	62.5 ± 4.3^*^	−2.2 ± 6.4

*Casearia sylvestris *	Leaves	26.5	62.5	58.0 ± 2.0^*^	12.1 ± 5.6^*^
			125	72.7 ± 0.7^*^	92.7 ± 0.9^*^
			250	76.6 ± 0.6^*^	95.5 ± 0.1^*^

*Chrysophyllum marginatum *	Aerial parts	14.7	62.5	49.0 ± 1.4^*^	−19.2 ± 5.1
			125	59.5 ± 0.6^*^	−19.3 ± 5.2
			250	79.1 ± 0.4^*^	3.8 ± 9.2

*Coccoloba cereifera *	Aerial parts	11.2	62.5	33.0 ± 3.4^*^	−7.6 ± 2.4
			125	49.5 ± 0.8^*^	−8.2 ± 0.4
			250	58.1 ± 0.4^*^	−7.1 ± 2.3

*Cuphea carthagenensis *	Aerial parts	11.1	62.5	25.7 ± 0.6^*^	−43.3 ± 2.4
			125	9.8 ± 4.8	−41.2 ± 1.2
			250	15.7 ± 3.0^*^	−14.0 ± 6.6

*Erythroxylum gonocladum *	Leaves	23.6	62.5	20.4 ± 9.8^*^	−3.2 ± 4.4
			125	11.0 ± 5.5	−1.8 ± 3.8
			250	25.5 ± 16.6^*^	51.2 ± 18.7^*^

*Erythroxylum suberosum *	Leaves	18.6	62.5	−22.2 ± 1.5	−12.2 ± 0.6
			125	−4.2 ± 1.4	−11.9 ± 0.9
			250	28.2 ± 7.5^*^	−12.9 ± 1.9

*Erythroxylum tortuosum *	Leaves	27.2	62.5	−49.3 ± 13.6	−29.5 ± 4.8
			125	−42.3 ± 7.2	−25.2 ± 2.9
			250	−21.5 ± 3.9	15.0 ± 19.8^*^

*Lippia sidoides *	Leaves	18.0	62.5	36.2 ± 5.7^*^	30.7 ± 6.5^*^
			125	41.6 ± 2.4^*^	77.5 ± 5.4^*^
			250	42.1 ± 0.1^*^	96.0 ± 0.1^*^

*Paepalanthus bromelioides *	Aerial parts	7.7	62.5	1.5 ± 5.7	−58.7 ± 9.5
			125	14.5 ± 2.2^*^	−51.2 ± 6.8
			250	30.0 ± 2.8^*^	−36.3 ± 7.5

*Phoenix roebelenii *	Aerial parts	14.7	62.5	28.0 ± 1.1^*^	−2.1 ± 9.8
			125	7.0 ± 3.80	−10.3 ± 9.2
			250	26.8 ± 6.3^*^	37.5 ± 3.8^*^

*Sida carpinifolia *	Aerial parts	8.7	62.5	29.2 ± 0.9^*^	−12.1 ± 2.9
			125	1.1 ± 3.1	−9.8 ± 2.4
			250	69.2 ± 0.6^*^	−8.9 ± 4.6

*Sida glaziovii *	Aerial parts	10.5	62.5	39.2 ± 1.2^*^	−46.1 ± 1.4
			125	39.6 ± 4.2^*^	−44.3 ± 3.1
			250	58.0 ± 3.9^*^	−29.1 ± 3.1

*Terminalia catappa *	Leaves	6.7	62.5	−51.0 ± 9.3	−33.0 ± 7.1
			125	−39.3 ± 9.2	−31.0 ± 5.3
			250	73.4 ± 0.6^*^	41.6 ± 14.9^*^

*Terminalia glabrescens *	Leaves	7.5	62.5	48.3 ± 1.8^*^	3.7 ± 1.1
			125	52.3 ± 1.8^*^	10.3 ± 2.1
			250	59.1 ± 1.6^*^	57.2 ± 2.3^*^

*Vellozia piresiana *	Leaves	9.8	62.5	15.7 ± 3.3^*^	−8.6 ± 6.2
			125	−5.8 ± 5.6	0.9 ± 5.2
			250	15.0 ± 1.4^*^	46.2 ± 9.5^*^

^a^Inflammatory mediators production (absolute values in pg/mL).

^*^Differences in the release of TNF-*α* and CCL-2 elicited by the extracts were analyzed by one-way ANOVA followed by multiple comparisons of Newman-Keuls. In each extract, ∗ indicates significant inhibition of TNF-*α* or CCL-2 release in comparison to LPS-stimulated cells (*P* < 0.05). Dexamethasone (0.1 *μ*M) was employed as positive control.

**Table 3:** IC_50_ values determined for selected extracts on *in vitro* inhibition of inflammatory mediators and radical scavenging activity.

Plant species	TNF-*α* (*µ*g/mL ± SD)	CCL2 (*µ*g/mL ± SD)	^*^DPPH assay (*µ*g/mL ± SD)	Inhibition ratio extract/rutin
*Barbacenia flava *	>250	>250	6.61 ± 1.7^f^	0.77
*Calophyllum brasiliense *	>250	>250	6.03 ± 2.2^g^	0.70
*Caryocar brasiliense *barks	>500	>250	6.13 ± 1.5^g^	0.71
*Caryocar brasiliense *leaves	224.0 ± 1.0	>250	3.01 ± 1.0^k^	0.35
*Casearia sylvestris *	124.0 ± 1.0	>250	5.70 ± 2.1^gh^	0.66
*Coccoloba cereifera *	194.3 ± 1.1	>250	4.12 ± 1.4^i^	0.48
*Cuphea carthagenensis *	>250	>250	5.40 ± 1.6^h^	0.62
*Chrysophyllum marginatum *	>500	>250	6.93 ± 1.3^f^	0.80
*Erythroxylum gonocladum *	>250	>250	8.81 ± 1.0^cd^	1.02
*Erythroxylum suberosum *	>250	>250	5.72 ± 2.0^gh^	0.66
*Erythroxylum tortuosum *	>250	>250	6.55 ± 1.0^f^	0.76
*Lippia sidoides *	>500	64.3 ± 1.0	7.56 ± 1.8^e^	0.88
*Paepalanthus bromelioides *	>250	>250	3.62 ± 1.4^j^	0.42
*Phoenix roebelenii *	>250	>250	6.56 ± 2.0^f^	0.76
*Sida carpinifolia *	>250	>250	13.23 ± 3.9^a^	1.54
*Sida glaziovii *	>250	>250	5.46 ± 2.4^h^	0.63
*Terminalia catappa *	>250	>250	6.12 ± 1.9^g^	0.71
*Terminalia glabrescens *	153.5 ± 1.1	189.4 ± 1.0	10.66 ± 1.9^b^	1.24
*Vellozia piresiana *	>250	>250	7.50 ± 4.2^e^	0.84
Rutin	ND	ND	8.58 ± 1.0^d^	1

ND: not determined.

^*^IC_50_ values were analyzed by one-way ANOVA, followed by Tukey's test. Values with different letters differ statistically from each other according to Tukey's test (*P* < 0.05).
